# Comparison of International Guidelines for Assessment of Suspected Stable Angina: Insights From the PROMISE and SCOT-HEART

**DOI:** 10.1016/j.jcmg.2018.06.021

**Published:** 2018-09

**Authors:** Philip D. Adamson, David E. Newby, C. Larry Hill, Adrian Coles, Pamela S. Douglas, Christopher B. Fordyce

**Affiliations:** a British Heart Foundation Centre for Cardiovascular Science, University of Edinburgh, Edinburgh, United Kingdom; b Duke Clinical Research Institute, Duke University School of Medicine, Durham, North Carolina; c Division of Cardiology, University of British Columbia, Vancouver, British Columbia, Canada

**Keywords:** clinical guidelines, coronary artery disease, coronary computed tomography angiography, stable angina, ACC, American College of Cardiology, AHA, American Heart Association, CAD, coronary artery disease, CCTA, coronary computed tomography angiography, CI, confidence interval, ESC, European Society of Cardiology, HR, hazard ratio, NICE, National Institute of Health and Care Excellence, OR, odds ratio, PTP, pre-test probability

## Abstract

**Objectives:**

This study sought to compare the performance of major guidelines for the assessment of stable chest pain including risk-based (American College of Cardiology/American Heart Association and European Society of Cardiology) and symptom-focused (National Institute for Health and Care Excellence) strategies.

**Background:**

Although noninvasive testing is not recommended in low-risk individuals with stable chest pain, guidelines recommend differing approaches to defining low-risk patients.

**Methods:**

Patient-level data were obtained from the PROMISE (Prospective Multicenter Imaging Study for Evaluation of Chest Pain) and SCOT-HEART (Scottish Computed Tomography of the Heart) trials. Pre-test probability was determined and patients dichotomized into low-risk and intermediate-high–risk groups according to each guideline’s definitions. The primary endpoint was obstructive coronary artery disease on coronary computed tomography angiography. Secondary endpoints were coronary revascularization at 90 days and cardiovascular death or nonfatal myocardial infarction up to 3 years.

**Results:**

In total, 13,773 patients were included of whom 6,160 had coronary computed tomography angiography. The proportions of patients identified as low risk by the American College of Cardiology/American Heart Association, European Society of Cardiology, and National Institute for Health and Care Excellence guidelines, respectively, were 2.5%, 2.5%, and 10.0% within PROMISE, and 14.0%, 19.8%, and 38.4% within SCOT-HEART. All guidelines identified lower rates of obstructive coronary artery disease in low- versus intermediate-high–risk patients with a negative predictive value of ≥0.90. Compared with low-risk groups, all intermediate-high–risk groups had greater risks of coronary revascularization (odds ratio [OR]: 2.2 to 24.1) and clinical outcomes (OR: 1.84 to 5.8).

**Conclusions:**

Compared with risk-based guidelines, symptom-focused assessment identifies a larger group of low-risk chest pain patients potentially deriving limited benefit from noninvasive testing. (Scottish Computed Tomography of the Heart Trial [SCOT-HEART]; NCT01149590; Prospective Multicenter Imaging Study for Evaluation of Chest Pain [PROMISE]; NCT01174550)

## Graphical abstract

**Figure:**
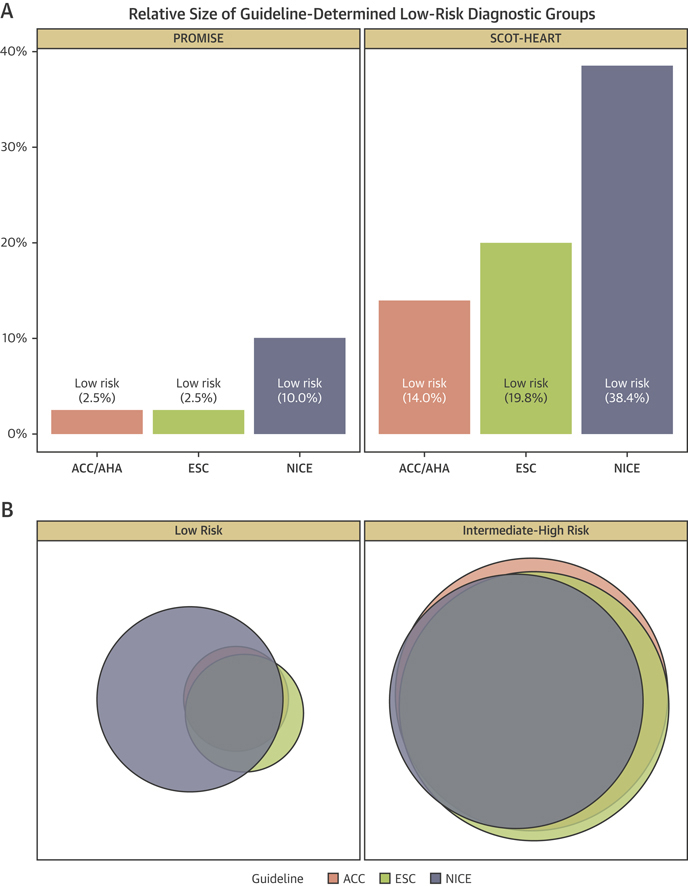

The safe and efficient assessment of individuals presenting with suspected stable angina is fraught with challenge. At an individual level, clinicians and patients alike are highly motivated to determine the cause of symptoms and identify the presence of underlying coronary artery disease (CAD) that may place the patient at high risk of future cardiovascular events. Given the resource-intensive nature of cardiac investigations, this tendency toward risk aversion must be balanced on a population level by efficient diagnostic pathways that minimize unnecessary or inappropriate testing.

Optimizing this balance of safety and efficiency underpins the principles of international clinical guidelines. In recent years, 3 distinct approaches have been independently adopted by the American College of Cardiology/American Heart Association (ACC/AHA) 1, 2, the European Society of Cardiology (ESC) (3), and the U.K. National Institute of Health and Care Excellence (NICE) 4, 5.

Both the ACC/AHA and ESC guidelines adopt the concept of Bayesian probability whereby initial estimation of prior probability is updated according to diagnostic test results to determine the post-test probability of obstructive CAD. Within these risk-based strategies, pre-test probability (PTP) is determined from the DF-CASS (Diamond-Forrester/Coronary Artery Surgery Study) (ACC/AHA) (2) and CADC (Coronary Artery Disease Consortium) (ESC) (3) clinical risk scores that incorporate age, sex, and chest pain typicality. Knowledge of PTP is used to categorize patients into 1 of 3 diagnostic risk groups: low; intermediate; or high. Both guidelines agree that noninvasive testing for CAD has greatest utility (Class I recommendation) in the intermediate-risk group, which is arbitrarily defined as 10% to 90% in the United States and 15% to 85% in Europe. In contrast, the recently updated NICE guidance for the diagnosis of suspected stable angina has abandoned this probabilistic approach in favor of a symptom-focused assessment (4). Following clinical evaluation, patients adjudged to have typical or atypical symptoms or an abnormal resting electrocardiogram are categorized into a possible angina group for whom additional noninvasive imaging with coronary computed tomography angiography (CCTA) is recommended. The remainder are classified as nonanginal, and no further testing is indicated.

However, the impact of these recommendations on the appropriate selection of patients for the application of these tests remains underexplored in prospective clinical trials. Indeed, while all 3 of the guidelines recognize the limited utility of diagnostic testing in low-risk individuals, each has adopted important differences in approach to defining this cohort. To our knowledge, no prior study has systematically compared the results of the 3 approaches to identify obstructive CAD and clinical outcomes. Thus, we studied the efficiency and safety of the 3 major guidelines for the diagnosis of obstructive CAD in patients with stable chest pain within the context of 2 recent large clinical studies—the North American, PROMISE (Prospective Multicenter Imaging Study for Evaluation of Chest Pain), and the SCOT-HEART (Scottish Computed Tomography of the Heart) trial.

## Methods

### Study cohorts

Patient-level data were obtained from the PROMISE and SCOT-HEART trial cohorts. These are prospective multicenter randomized controlled trials investigating the utility of CCTA in the diagnosis and management of patients undergoing assessment of suspected stable angina due to CAD. The pragmatic designs 6, 7 and principal findings 8, 9 of these studies have been reported previously. The intervention arm in both studies consisted of CCTA, which was compared with usual care. Details of cohort-specific inclusion and exclusion criteria have been previously described (10). To confirm guideline utility in distinct clinical settings and across populations, the study cohorts were analyzed separately.

### Guideline-determined diagnostic groups

For the ACC/AHA and ESC guideline analysis, PTP of CAD was determined according to the DF-CASS and CADC risk models, respectively. Diagnostic risk groups (low, intermediate, high) were then defined as specified in each guideline (Online Table 1). For the purposes of this analysis, we have combined the intermediate- and high-risk patients into a single intermediate-high–risk diagnostic group who are likely to require further diagnostic testing. For the NICE guideline analysis, patients with nonanginal symptoms and a normal resting electrocardiogram were classified as low risk with the remainder categorized as intermediate-high risk.

### Coronary imaging

Patients randomized to the intervention arms of both trials underwent cardiac imaging with contrast-enhanced CCTA using a 64-slice or greater multidetector CT scanner. The presence of obstructive CAD was defined as site interpretation of ≥70% area stenosis in any major epicardial vessel or ≥50% stenosis in the left main stem. In concordance with the ACC/AHA guideline, we additionally determined the presence of prognostically significant CAD, defined as 3-vessel disease, 2-vessel disease including the proximal left anterior descending artery, or obstructive disease involving the left main stem.

### Endpoints

The primary (diagnostic) endpoint was the presence of obstructive CAD on coronary imaging in those individuals randomized to the CCTA intervention arm who underwent this test as part of the initial trial protocols.

The secondary endpoints were determined from the entire study cohort of both trials and included coronary revascularization at 90 days—either coronary artery bypass grafting or percutaneous coronary intervention—and cardiovascular death or non-fatal myocardial infarction up to 3 years. The time point of 90 days reflects the duration of follow-up for this endpoint within the PROMISE trial and was chosen to capture CCTA-driven alterations in coronary revascularization. Longer-term outcome data for fatal and nonfatal cardiovascular events was recorded in all patients up to 1 year in PROMISE and up to 3 years in SCOT-HEART.

### Statistical analysis

Statistical analysis was performed using R (version 3.4.3, R Foundation for Statistical Computing, Vienna, Austria) and SAS (version 9.4, SAS Institute, Inc., Cary, North Carolina). All analyses were post hoc and were stratified by study cohort and according to intention-to-treat, irrespective of compliance with scanning. The diagnostic and revascularization endpoints were analyzed using chi-squared tests and log-binomial regression 11, 12, with results are reported as odds ratios (ORs) with 95% confidence intervals (CIs) and p values. Clinical events were analyzed with Cox regression and reported as hazard ratios (HRs) with cumulative incidence plots constructed. Additional performance measures were determined including discrimination, sensitivity, specificity, positive predictive value, and negative predictive value. In PROMISE, these performance measures were assessed 1 year post-randomization using the method of Heagerty et al. (13) to account for those lost to follow-up in the trial. In addition to these stratum-specific analyses, we modeled interaction terms for allocation and within study cohort to provide hypothesis testing for interaction on the relative scale. Comparison of diagnostic metrics including predictive values between the overlapping groups of patients determined to be low risk by each of the 3 guidelines were made using previously described methods 14, 15, 16, 17. Net reclassification improvement was compared between the NICE guideline and both ACC/AHA and ESC guidelines (18). All primary and secondary endpoints are reported unadjusted. Data are presented as mean ± SD or mean differences with 95% CI. Statistical significance was taken as 2-sided p < 0.05.

## Results

### Description of study cohorts

**Figure 1:**
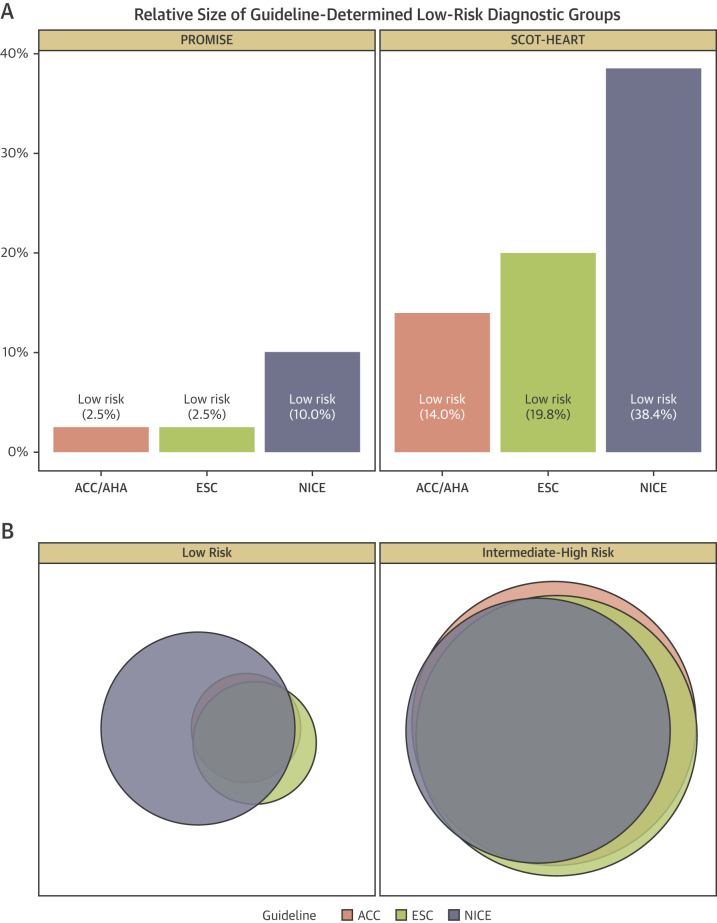
Diagnostic Group Classification According to the ACC/AHA, ESC, and NICE Guidelines The American College of Cardiology/American Heart Association (ACC/AHA), European Society of Cardiology (ESC), and National Institute of Health and Care Excellence (NICE) guidelines are **pink, green,** and **blue,** respectively. **(A)** Proportion of study population classified as low risk. **(B)** Overlap of diagnostic groups. Within this Euler diagram, the **area of each shaded circle** represents the proportion of patients classified into the low- and high-risk groups in the left- and right-hand panels, respectively. The **combined area of circles of the same color** represents the entire analysis population and is the same for all 3 colors corresponding to all 3 guidelines. **Overlapping areas** correspond to individual patients who fulfill the criteria for low or high risk according to more than 1 guideline. PROMISE = Prospective Multicenter Imaging Study for Evaluation of Chest Pain; SCOT-HEART = Scottish Computed Tomography of the Heart.

The PROMISE study population comprised 10,003 patients (age 61 ± 8 years, 53% female) without known CAD of whom 4,541 had interpretable CCTA results available. The SCOT-HEART population included 3,770 patients overall (376 patients excluded with known CAD) (57 ± 10 years, 46% female) of whom 1,619 had CCTA results available. The number of patients identified as low risk by the ACC/AHA, ESC, and NICE guidelines, respectively, were 250 (2.5%), 251 (2.5%), and 1,002 (10.0%) within PROMISE, and 528 (14.0%), 748 (19.8%), and 1,447 (38.4%) within SCOT-HEART (Table 1). Within both the SCOT-HEART and PROMISE trial populations, there was substantial overlap in individual patients identified as low risk by ACC/AHA and ESC with 486 (SCOT-HEART) and 250 (PROMISE) patients classified as low risk by both guidelines and only 42 (SCOT-HEART) and 1 (PROMISE) patients deemed low risk by ACC/AHA were considered intermediate-high risk by ESC. In contrast, there were 1,001 (SCOT-HEART) and 763 (PROMISE) patients defined as low risk by NICE who were classified as intermediate-high risk by either ACC/AHA or ESC (Figure 1).

**Table 1:** Baseline Characteristics by Guideline Risk Levels

	ACC/AHA (2012)		ESC (2013)		NICE (2016)		Complete Trial Cohort
	Low Risk	High Risk	Low Risk	High Risk	Low Risk	High Risk	
PROMISE							
Patients	250	9,753	251	9,752	1,002	9,001	10,003
Age, yrs	55.0 (52.5–57.7)	60.3 (54.6–66.2)	55.0 (52.4–57.7)	60.3 (54.6–66.2)	60.3 (54.3–66.5)	59.9 (54.5–65.9)	60.0 (54.4–65.9)
Female	250 (100.0)	5,020 (51.5)	251 (100.0)	5,019 (51.5)	564 (56.3)	4,706 (52.3)	5,270 (52.7)
BMI, kg/m^2^	30.5 (25.8–35.0)	29.7 (26.4–33.9)	30.4 (25.8–35.0)	29.7 (26.4–33.9)	29.3 (26.0–33.5)	29.7 (26.4–34.0)	29.7 (26.3–33.9)
Hypertension	152 (60.8)	6,349 (65.1)	153 (61.0)	6,348 (65.1)	611 (61.0)	5,890 (65.4)	6,501 (65.0)
Hypercholesterolemia	156 (62.4)	6,611 (67.8)	157 (62.5)	6,610 (67.8)	654 (65.3)	6,113 (67.9)	6,767 (67.7)
Diabetes mellitus	39 (15.6)	2,105 (21.6)	39 (15.5)	2,105 (21.6)	179 (17.9)	1,965 (21.8)	2,144 (21.4)
Smoking history, current/ex	119 (47.6)	4,985 (51.1)	119 (47.4)	4,985 (51.1)	491 (49.0)	4,613 (51.3)	5,104 (51.0)
PAD or cerebrovascular disease	7 (2.8)	545 (5.6)	7 (2.8)	545 (5.6)	46 (4.6)	506 (5.6)	552 (5.5)
Family history	83 (33.3)	3,119 (32.1)	83 (33.2)	3,119 (32.1)	296 (29.6)	2,906 (32.4)	3,202 (32.1)
Anginal symptoms							
Nonanginal	250 (100)	814 (8.3)	250 (99.6)	814 (8.3)	1,002 (100)	62 (0.7)	1,064 (10.6)
Atypical angina	0 (0)	7,773 (79.7)	1 (0.4)	7,772 (79.7)	0 (0)	7,773 (86.4)	7,773 (77.7)
Typical angina	0 (0)	1,116 (12.0)	0 (0)	1,166 (12.0)	0 (0)	1,166 (13.0)	1,166 (11.7)
Framingham 10-year CVD risk	8.7 (5.9–12.9)	17.4 (10.8–28.9)	8.7 (5.8–12.9)	17.4 (10.8–28.9)	15.6 (9.8–26.0)	17.3 (10.6–28.9)	17.1 (10.6–28.6)
SCOT-HEART							
Patients	528	3,242	748	3,022	1,447	2,323	3,770
Age, yrs	50.0 (42.0–54.0)	59.0 (51.0–65.0)	51.0 (46.0–58.0)	59.0 (51.0–66.0)	54.0 (47.0–61.0)	59.0 (52.0–66.0)	57.0 (50.0–64.0)
Female	465 (88.1)	1,256 (38.7)	727 (97.2)	994 (32.9)	669 (46.2)	1,052 (45.3)	1,721 (45.6)
BMI, kg/m^2^	29.2 (25.0–34.5)	28.7 (25.7–32.5)	29.1 (25.0–34.6)	28.7 (25.7–32.4)	28.4 (25.1–32.7)	29.0 (25.9–32.9)	28.8 (25.6–32.8)
Hypertension	112 (21.5)	1,099 (34.2)	179 (24.2)	1,032 (34.4)	362 (25.3)	849 (36.8)	1,211 (32.4)
Hypercholesterolemia	176 (33.3)	1,902 (58.7)	284 (38.0)	1,794 (59.4)	580 (40.1)	1,498 (64.5)	2,078 (55.1)
Diabetes mellitus	38 (7.2)	332 (10.2)	58 (7.8)	312 (10.3)	115 (7.9)	255 (11.0)	370 (9.8)
Smoking history, current/ex	270 (51.1)	1,684 (52.0)	384 (51.3)	1,570 (52.0)	736 (50.9)	1,218 (52.5)	1,954 (51.9)
PAD or cerebrovascular disease	14 (2.7)	150 (4.7)	22 (3.0)	142 (4.7)	52 (3.6)	112 (4.8)	164 (4.4)
Family history	251 (47.8)	1,307 (40.7)	353 (47.6)	1,205 (40.3)	580 (40.6)	978 (42.4)	1,558 (41.7)
Anginal symptoms							
Nonanginal	528 (100.0)	1,088 (33.6)	642 (85.8)	974 (32.2)	1,447 (100.0)	169 (7.3)	1,616 (42.9)
Atypical angina	0 (0.0)	893 (27.5)	106 (14.2)	787 (26.0)	0 (0.0)	893 (38.4)	893 (23.7)
Typical angina	0 (0.0)	1261 (38.9)	0 (0.0)	1261 (41.7)	0 (0.0)	1,261 (54.3)	1,261 (33.4)
Framingham 10-yr CVD risk	6.3 (3.6–9.3)	16.2 (10.0–25.2)	7.4 (4.2–11.2)	16.8 (10.3–25.9)	11.2 (6.6–18.2)	16.7 (9.6–27.0)	14.3 (8.4–23.5)

Values are n, median (interquartile range), n (%), mean ± SD.

ACC/AHA = American College of Cardiology/American Heart Association; BMI = body mass index; CVD = cerebrovascular disease; ESC = European Society of Cardiology; NICE = National Institute of Health and Care Excellence; PAD = peripheral arterial disease; PROMISE = Prospective Multicenter Imaging Study for Evaluation of Chest Pain; SCOT-HEART = Scottish Computed Tomography of the Heart.

### Prevalence of CAD by diagnostic groups

Overall, obstructive CAD was identified in 537 patients (11.8%) in PROMISE and 359 (22.2%) in SCOT-HEART. The prevalence of obstructive CAD was <10% in the low-risk groups as determined by all 3 guidelines within both study cohorts (Table 2), and across both trials, the number of patients with prognostically significant CAD in the low- versus intermediate-high–risk groups, respectively, was 1.3% versus 4.6% (ACC/AHA), 1.5% versus 4.7% (ESC), and 2.4% versus 4.9% (NICE). For all comparisons, the OR for CAD was significantly lower in the low-risk than in intermediate-high–risk groups (p < 0.05 for all) (Table 3). Compared with the ACC/AHA and ESC definitions, respectively, applying the NICE criteria resulted in a 12.6% and 10.6% net increase in the proportion of patients without obstructive CAD appropriately identified as low risk. There was a smaller net increase in the number of patients with CAD inappropriately classified as low risk (9.3% [ACC/AHA] and 8.6% [ESC]) (Online Table 2). In comparison with both the ACC/AHA and ESC classifications, the determination of low risk according to the NICE guideline was associated with greater diagnostic specificity (p < 0.001 for both) at the expense of a decrease in sensitivity (p < 0.001 for both).

**Table 2:** Patient Outcomes by Guideline Risk Levels

	ACC (2012)		ESC (2013)		NICE (2016)	
	Low Risk	High Risk	Low Risk	High Risk	Low Risk	High Risk
PROMISE						
Patients	250	9,753	251	9,752	1,002	9,001
CAD on CCTA	108	4,433	108	4,433	456	4,085
Normal	55 (50.9)	1,463 (33.0)	55 (50.9)	1,463 (33.0)	155 (34.0)	1,363 (33.4)
Mild CAD	50 (46.3)	2,436 (55.0)	50 (46.3)	2,436 (55.0)	262 (57.5)	2,224 (54.4)
Obstructive CAD	3 (2.8)	534 (12.0)	3 (2.8)	534 (12.0)	39 (8.6)	498 (12.2)
Prognostically significant CAD∗	0 (0)	144 (3.2)	0 (0)	144 (3.2)	11 (2.4)	133 (3.3)
Subsequent coronary revascularization—90 days	3 (1.2)	468 (4.8)	3 (1.2)	468 (4.8)	23 (2.3)	448 (5.0)
PCI	3 (1.2)	361 (3.7)	3 (1.2)	361 (3.7)	22 (2.2)	342 (3.8)
CABG	0 (0.0)	107 (1.1)	0 (0.0)	107 (1.1)	1 (0.1)	106 (1.2)
CVD death or nonfatal MI—1 yr	2 (0.8)	155 (1.6)	2 (0.8)	155 (1.6)	9 (0.9)	148 (1.6)
SCOT-HEART						
Patients	528	3,242	748	3,022	1,447	2,323
CAD on CCTA	193	1,426	305	1,314	591	1,028
Normal	138 (71.5)	498 (34.9)	198 (64.9)	438 (33.3)	296 (50.1)	340 (33.1)
Mild CAD	46 (23.8)	578 (40.5)	92 (30.2)	532 (40.5)	239 (40.4)	385 (37.5)
Obstructive CAD	9 (4.7)	350 (24.5)	15 (4.9)	344 (26.2)	56 (9.5)	303 (29.5)
Prognostically significant CAD∗	4 (2.1)	127 (8.9)	6 (2.0)	125 (9.5)	12 (2.0)	119 (11.6)
Subsequent coronary revascularization—90 days	2 (0.4)	249 (7.7)	4 (0.5)	247 (8.2)	7 (0.5)	244 (10.5)
PCI	2 (0.4)	217 (6.7)	4 (0.5)	215 (7.1)	7 (0.5)	212 (9.1)
CABG	0 (0.0)	33 (1.0)	0 (0.0)	33 (1.1)	0 (0.0)	33 (1.4)
CVD death or nonfatal MI—3 yrs	2 (0.4)	71 (2.2)	4 (0.5)	69 (2.3)	17 (1.2)	56 (2.4)

Values are n or n (%).

CABG = coronary artery bypass graft; CAD = coronary artery disease; CCTA = coronary computed tomography angiography; MI = myocardial infarction; PCI = percutaneous coronary intervention; other abbreviations as in Table 1.

∗ Prognostically significant CAD was defined as 3-vessel disease, 2-vessel disease including the proximal left anterior descending artery, or obstructive disease involving the left main stem.

**Table 3:** Association Between Guideline Risk Level and Obstructive CAD by Guideline

Guideline (Year)	Obstructive CAD (Events/Sample Size)		Unadjusted∗		Performance Measures				
	High Risk n/n (%)	Low Risk n/n (%)	OR (95% CI)	p Value	C Statistic (95% CI)	Sensitivity (95% CI)	Specificity (95% CI)	PPV (95% CI)	NPV (95% CI)
PROMISE									
ACC/AHA (2012)	534/4,433 (12.05)	3/108 (2.78)	4.79 (1.52–15.16)	0.008	0.510 (0.506–0.514)	0.994 (0.984–0.999)	0.026 (0.022–0.032)	0.120 (0.111–0.130)	0.972 (0.921–0.994)
ESC (2013)	534/4,433 (12.05)	3/108 (2.78)	4.79 (1.52–15.16)	0.008	0.510 (0.506–0.514)	0.994 (0.984–0.999)	0.026 (0.022–0.032)	0.120 (0.111–0.130)	0.972 (0.921–0.994)
NICE (2016)	498/4,085 (12.19)	39/456 (8.55)	1.48 (1.06–2.09)	0.023	0.516 (0.504–0.528)	0.927 (0.902–0.948)	0.104 (0.095–0.114)	0.122 (0.112–0.132)	0.914 (0.885–0.939)
SCOT-HEART									
ACC/AHA (2012)	350/1,426 (24.5)	9/193 (4.7)	6.65 (3.37–13.13)	<0.001	0.560 (0.548–0.573)	0.975 (0.966–0.981)	0.146 (0.130–0.164)	0.245 (0.225–0.267)	0.953 (0.942–0.963)
ESC (2013)	344/1,314 (26.2)	15/305 (4.9)	6.86 (4.02–11.69)	<0.001	0.594 (0.579–0.610)	0.958 (0.947–0.967)	0.230 (0.210–0.251)	0.262 (0.241–0.284)	0.951 (0.939–0.960)
NICE (2016)	303/1,028 (29.5)	56/591 (9.5)	3.99 (2.94–5.42)	<0.001	0.634 (0.611–0.658)	0.844 (0.826–0.861)	0.425 (0.401–0.449)	0.295 (0.273–0.317)	0.905 (0.890–0.919)

CI = confidence interval; NPV = negative predictive value; OR = odds ratio; PPV = positive predictive value; other abbreviations as in Table 1.

∗ Unadjusted model contains referral to guideline risk level (intermediate/high vs. low).

### Revascularization by diagnostic groups

During the first 90 days following randomization, 469 patients (4.7%) in PROMISE and 251 (6.7%) in SCOT-HEART underwent coronary revascularization procedures. Across both trials, the frequencies of revascularization within the low- versus intermediate-high–risk groups were as follow: ACC/AHA, 5 (0.7%) versus 717 (5.5%) (OR: 8.6; 95% CI: 3.6 to 20.8); ESC, 7 (0.7%) versus 715 (5.6%) (OR: 8.0; 95% CI: 3.8 to 16.9); and NICE, 30 (2.0%) versus 692 (6.9%) (OR: 3.5; 95% CI: 2.4 to 5.1) (p < 0.01 for all comparisons) (Table 4). In both trial cohorts, identification as low risk was associated with a negative predictive value for coronary revascularization of >0.97 for each of the 3 guidelines that was the same irrespective of guideline adopted (p > 0.05 for all). In contrast, the positive predictive value for coronary revascularization of the NICE classification was greater than either of the other guidelines (p < 0.001 for all).

**Table 4:** Association Between Guideline Risk Level and Revascularization Within 90 Days of Randomization

Guideline (Year)	Frequency of Revascularization (Events/Sample Size)		Unadjusted∗		Performance Measures				
	High Risk n/n (%)	Low Risk n/n (%)	OR (95% CI)	p Value	C Statistic (95% CI)	Sensitivity (95% CI)	Specificity (95% CI)	PPV (95% CI)	NPV (95% CI)
PROMISE									
ACC/AHA (2012)	466/9,753 (4.78)	3/250 (1.20)	4.13 (1.32–12.95)	0.015	0.510 (0.506–0.514)	0.994 (0.981–0.999)	0.026 (0.023–0.029)	0.048 (0.044–0.052)	0.988 (0.965–0.998)
ESC (2013)	466/9,752 (4.78)	3/251 (1.20)	4.15 (1.32–13.00)	0.015	0.510 (0.506–0.514)	0.994 (0.981–0.999)	0.026 (0.023–0.029)	0.048 (0.044–0.052)	0.988 (0.966–0.998)
NICE (2016)	446/9,001 (4.96)	23/1,002 (2.30)	2.22 (1.45–3.39)	<0.001	0.527 (0.517–0.537)	0.951 (0.927–0.969)	0.103 (0.097–0.109)	0.050 (0.045–0.054)	0.977 (0.966–0.985)
SCOT-HEART									
ACC/AHA (2012)	249/3,242 (7.7)	2/528 (0.4)	21.88 (5.43–88.25)	<0.001	0.571 (0.563–0.579)	0.992 (0.989–0.994)	0.149 (0.138–0.161)	0.077 (0.069–0.086)	0.996 (0.994–0.998)
ESC (2013)	247/3,022 (8.2)	4/748 (0.5)	16.56 (6.15–44.59)	<0.001	0.598 (0.587–0.608)	0.984 (0.980–0.988)	0.211 (0.199–0.225)	0.082 (0.073–0.091)	0.995 (0.992–0.997)
NICE (2016)	244/2,323 (4.8)	7/1,447 (0.5)	24.14 (11.36–51.34)	<0.001	0.691 (0.678–0.704)	0.972 (0.966–0.977)	0.409 (0.394–0.425)	0.105 (0.096–0.115)	0.995 (0.992–0.997)

Abbreviations as in Tables 1 and 3.

∗ Unadjusted model contains referral to guideline risk level (intermediate/high vs. low).

### Cardiovascular death or nonfatal myocardial infarction by diagnostic groups

During follow-up, 157 patients (1.6%) in PROMISE and 73 (1.9%) in SCOT-HEART experienced a nonfatal myocardial infarction or died from a cardiovascular cause. The incidence rates per 100 patient-years within PROMISE for the low- versus intermediate-high–risk groups were as follow: ACC/AHA, 0.39 versus 0.78 (HR: 2.0; 95% CI: 0.5 to 8.1; p = 0.330); ESC, 0.39 versus 0.78 (HR: 2.0; 95% CI: 0.5 to 8.1; p = 0.326); and NICE, 0.43 versus 0.81 (HR: 1.84; 95% CI: 0.9 to 3.6; p = 0.076). The incidence rates within SCOT-HEART for the low- versus intermediate-high–risk groups were as follow: ACC/AHA, 0.12 versus 0.67 (HR: 5.8; 95% CI: 1.4 to 23.8; p = 0.014); ESC, 0.16 versus 0.70 (HR: 4.3; 95% CI: 1.6 to 11.8; p = 0.005); and NICE, 0.36 versus 0.73 (HR: 2.1; 95% CI: 1.2 to 3.6; p = 0.009) (Table 5). On analysis of both trial cohorts in combination, although the negative predictive value for cardiovascular death or nonfatal myocardial infarction was lower when applying the NICE classification than either the ACC/AHA (p = 0.034) or ESC (p = 0.047) strategies, it remained >0.98 for each of the 3 guidelines.

**Table 5:** Association Between Guideline Risk Level and CVD Death/MI

Guideline (Year)	Incidence Rate per 100 Patient-Years		Unadjusted∗	Performance Measures					
	High Risk (95% CI)	Low Risk (95% CI)	HR (95% CI)	p Value	C Statistic (95% CI)	Sensitivity (95% CI)	Specificity (95% CI)	PPV (95% CI)	NPV (95% CI)
PROMISE									
ACC/AHA (2012)	0.78 (0.67–0.92)	0.39 (0.10–1.55)	2.00 (0.50–8.07)	0.330	0.508 (0.499–0.516)	0.987 (0.955–0.999)	0.025 (0.022–0.029)	0.016 (0.014–0.019)	0.992 (0.971–0.999)
ESC (2013)	0.78 (0.67–0.92)	0.39 (0.10–1.54)	2.01 (0.50–8.12)	0.326	0.508 (0.499–0.516)	0.987 (0.955–0.999)	0.025 (0.022–0.028)	0.016 (0.014–0.019)	0.992 (0.972–0.999)
NICE (2016)	0.81 (0.69–0.95)	0.43 (0.23–0.83)	1.84 (0.94–3.61)	0.076	0.524 (0.505–0.543)	0.943 (0.894–0.974)	0.101 (0.095–0.107)	0.016 (0.014–0.019)	0.991 (0.983–0.996)
SCOT-HEART									
ACC/AHA (2012)	0.67 (0.52–0.84)	0.12 (0.01–0.42)	5.85 (1.44–23.85)	0.014	0.557 (0.538–0.577)	0.973 (0.967–0.977)	0.142 (0.131–0.154)	0.022 (0.018–0.027)	0.996 (0.994–0.998)
ESC (2013)	0.70 (0.54–0.88)	0.16 (0.04–0.42)	4.31 (1.57–11.80)	0.005	0.573 (0.546–0.600)	0.945 (0.937–0.952)	0.201 (0.189–0.214)	0.023 (0.019–0.028)	0.995 (0.992–0.997)
NICE (2016)	0.73 (0.55–0.95)	0.36 (0.21–0.58)	2.07 (1.20–3.56)	0.009	0.577 (0.528–0.626)	0.767 (0.753–0.780)	0.387 (0.371–0.402)	0.024 (0.020–0.030)	0.988 (0.984–0.991)

HR = hazard ratio; other abbreviations as in Tables 1 and 2.

∗ Unadjusted model contains referral to guideline risk level (intermediate/high vs. low).

## Discussion

Identifying which patients require additional testing is a central component of the care of stable symptomatic patients with suspected CAD. Because the approach to doing so and resulting recommendations differ across the 3 major international guidelines, which together represent the current standard of care across Europe and North America, we compared their application within 2 large, geographically distinct, randomized trial populations. Despite the substantial demographic and clinical practice differences between these cohorts, we have demonstrated proportionally consistent findings. In both trials, the use of a symptom-focused strategy endorsed by NICE, in contrast with a Bayesian–risk based approach endorsed by ACC/AHA and ESC, resulted in a 3- to 4-fold increase in the number of patients for whom no further investigation for the presence of CAD is recommended. This is reassuring given concerns raised recently that the updated NICE guidance would lead to an increase in indiscriminate diagnostic testing (19), as well as strategies being considered to defer testing in those patients with very low risk 20, 21. Furthermore, the group designated by NICE for no testing (nonanginal symptoms) demonstrated <10% prevalence of coronary obstruction across both trial cohorts, below the threshold adjudged to reflect low risk in both the ACC/AHA and ESC guidelines. These findings strongly support the use of characterization of patient symptoms as central in the assessment of suspected stable angina.

Recently, in part prompted by rapid advances in coronary CT, extensive research has been undertaken to clarify the relative merits of noninvasive imaging modalities in the assessment of suspected stable angina. Indeed, both PROMISE and SCOT-HEART were designed to test the hypothesis that CCTA might improve clinical outcomes compared with established, usual care approaches. In this context, CCTA increases diagnostic certainty and may reduce cardiovascular events 8, 9, 22.

In practice however, it is well appreciated that many patients undergoing assessment for possible stable angina are at low risk of both underlying CAD and future ischemic events, at least in the short to medium term. In the primary care setting, <10% of such patients are ultimately identified as having a coronary cause for their symptoms (23). Recognizing this, substantial work has been done to update, refine, and extend risk models for estimating the PTP CAD, albeit often in highly selected populations referred for invasive angiography 24, 25, 26, 27, 28. In stark contrast, there is a dearth of trial evidence to support the clinical efficacy of the risk thresholds recommended within the guidelines and it seems plausible that although symptom characterization has recognized value in all these guidelines, its importance continues to be undervalued (29). Temporal trends described in patients undergoing nuclear testing offer valuable insight in this regard. In a report of nearly 40,000 patients covering the period 1991 to 2009, the proportion of patients referred for myocardial perfusion imaging with inducible ischemia fell from 30% to 5% (30). This occurred despite increasing prevalence of cardiovascular risk factors and a corresponding increase in the calculated PTP of CAD within this cohort. Similar declining rates of positive ischemia tests have been described elsewhere 31, 32. Interestingly, over the same time period, the proportion of these patients with typical angina symptoms fell from 13% to 2%, whereas those reporting only dyspnea in the absence of chest discomfort, increased from 5% to 11% (30). This association places further emphasis on the need for accurate symptom characterization to lie at the center of decision making and is entirely consistent with our analysis, wherein we identified a substantial increase in diagnostic specificity when applying the symptom-focused NICE guideline compared with either of the alternative approaches.

Although our findings were proportionally similar across both study cohorts, important differences in trial design likely explain the differences in overall percentages of patients within each of the diagnostic groups. The trial inclusion criteria in PROMISE stipulated that physicians had predetermined a requirement for noninvasive testing, whereas SCOT-HEART enrolled all patients referred to the chest pain clinic, irrespective of clinical gestalt. This may account for why individuals presenting with chest pain classified as nonanginal comprised merely 11% within PROMISE compared with 41% in SCOT-HEART. In contrast, only 72% of patients within PROMISE described chest pain as the primary symptom compared with the entire SCOT-HEART cohort (10), perhaps explaining the 2-fold greater prevalence of obstructive CAD in the latter, despite comprising a population at apparently lower cardiovascular risk, as determined by the Framingham score.

Whereas a Bayesian probabilistic approach to patient selection has many theoretical advantages, our results point toward a key limitation in this strategy. Namely, that despite revisions, risk models continue to both over- and underestimate disease prevalence 9, 33, 34, 35, 36, 37, 38 when applied in settings external to the derivation cohort. Given it is the presence of symptoms that identifies patients with suspected angina, it would appear to follow that it is the nature of these symptoms that should inform diagnostic decisions. This perhaps explains why removing cardiovascular risk factors—namely age and sex—that are common to both the ACC/AHA and ESC guidelines, results in the NICE guideline’s improved diagnostic discrimination (39). Crucially, this approach appears safe, as the prevalence of CAD remained below 10% among patients with nonanginal symptoms in both trial cohorts.

### Study strengths and limitations

Our study has several notable strengths. Both the PROMISE and SCOT-HEART trials were pragmatic in design and enrolled patients that accurately reflect the real-world suspected angina population. Despite its post hoc nature, this analysis combines the 2 largest prospective trials of CCTA for stable angina to date, and the enrolled sample size and clinical and geographic diversity of these study cohorts provides robust evidence that our findings are applicable across international boundaries and in a variety of clinical settings. In both cases, patient characteristics regarding symptoms, cardiovascular risk factors, noninvasive test results and clinical endpoints were collected in a systematic manner with minimal loss to follow-up. Any minor differences between trials in data collection are minimized because all analyses comparing guidelines were conducted within each trial rather than in combined data. We chose to combine both intermediate- and high-risk individuals into a single intermediate-high–risk category, a necessary deviation from ACC/AHA and ESC guideline recommendations, to allow comparison of those who do versus do not need testing and to allow direct comparison with the 2 diagnostic groups created by the NICE guideline. Although CCTA has limitations in the diagnosis of CAD, these principally relate to suboptimal specificity and a tendency to overestimate stenosis severity. In contrast, the very high diagnostic sensitivity offered by CCTA provides necessary reassurance regarding the ability of all strategies to exclude significant CAD. Importantly, the disease prevalence identified is unlikely to be an underestimate, and in fact the rates of CAD may be lower than we have reported in both the low- and high-risk diagnostic groups.

## Conclusions

All 3 current guidelines identify low-risk groups in both PROMISE and SCOT-HEART who have lower prevalence of CAD, including prognostically significant CAD, as well as fewer revascularizations and adverse events. Compared with traditional, risk-based guidelines, a symptom-focused strategy classifies a greater proportion of chest pain patients as low risk. Using this strategy has the potential to substantially reduce the use of downstream investigations in the diagnosis of suspected stable angina. These results suggest that a symptom-focused assessment may safely and efficiently identify low-risk patients deriving limited benefit from noninvasive testing.

Perspectives **COMPETENCY IN MEDICAL KNOWLEDGE:** In the assessment of suspected stable angina, noninvasive diagnostic imaging is recommended for intermediate-risk patients.

**COMPETENCY IN PATIENT CARE AND PROCEDURAL SKILLS:** Compared with traditional risk-based approaches, greater emphasis on symptomatology following careful clinical history taking can safely identify a greater number of patients at low risk of CAD and may reduce the requirement for additional investigations.

**TRANSLATIONAL OUTLOOK 1:** Updated clinical guidelines should place greater emphasis on the importance of patient symptoms in identifying appropriate individuals for diagnostic testing.

**TRANSLATIONAL OUTLOOK 2:** The clinical outcomes arising from international guidelines should be robustly evaluated to ensure they achieve optimal safety and efficacy.
