# Supplementary material for: Comparison of International Guidelines for Assessment of Suspected Stable Angina: Insights From the PROMISE and SCOT-HEART
Source: JACC Cardiovasc Imaging. 2018 Sep;11(9):1301–10. doi: 10.1016/j.jcmg.2018.06.021 (PMC6130226; doi:10.1016/j.jcmg.2018.06.021)
Supplement: Online Tables 1 and 2 [file mmc1.docx]

**Supplementary Appendix**

**Comparison of international guidelines for assessment of suspected stable angina: Insights from PROMISE and SCOT-HEART**

### Brief Title:

Assessment of stable angina: comparison of guidelines

### Authors:

Philip D. Adamson^1^ MD, PhD, David E. Newby^1^ MD, PhD, C. Larry Hill^2^ PhD, Adrian Coles^2^ PhD, Pamela S. Douglas^2^ MD, Christopher B. Fordyce^3^ MD MHS MSc

### Affiliations:

^1^BHF Centre for Cardiovascular Science, University of Edinburgh, Edinburgh, United Kingdom,

^2^Duke Clinical Research Institute, Duke University School of Medicine, Durham, North Carolina

^3^Division of Cardiology, University of British Columbia, Vancouver, British Columbia, Canada

### Correspondence to:

Dr. Philip D. Adamson

Room SU 305

BHF Centre for Cardiovascular Science

Chancellor’s Building

University of Edinburgh

49 Little France Cres

Edinburgh

EH16 4SB

UNITED KINGDOM

Email: [philip.adamson@ed.ac.uk](mailto:philip.adamson@ed.ac.uk)

| **Guideline** |  |  |
| --- | --- | --- |
|  | **Low-Risk** | **Intermediate-High Risk** |
| **American College of Cardiology/****American Heart Association** | Pre-test probability of CAD* <10% | Pre-test probability of CAD* ≥10% |
| **European Society of Cardiology** | Pre-test probability of CAD^†^ <15% | Pre-test probability of CAD^†^ ≥15% |
| **National Institute for Health and Care Excellence** | Non-anginal discomfort^‡^ AND normal resting ECG | Atypical angina^‡^ OR  Typical angina^‡^ OR  Non-anginal symptoms AND abnormal resting ECG |

**Supplementary Table 1: Classification of patients at low-risk and intermediate-high risk for coronary artery disease**

CAD, coronary artery disease; ECG, electrocardiogram.

*Pre-test probability determined according to the Diamond-Forrester/Coronary Artery Surgery Study risk score (2)

^†^Pre-test probability determined according to the Coronary Artery Disease Consortium risk score (3)

^‡^Symptom typicality with the NICE guideline has been adapted from the Diamond classification of chest discomfort (40). Typical angina is defined as all 3 of: 1) constricting discomfort in the front of the chest, or in the neck, shoulders, jaw, or arms; 2) precipitated by physical exertion; 3) relieved by rest or GTN within about 5 minutes. Atypical angina fulfills 2 of these criteria and non-anginal chest discomfort fulfills ≤1 of these criteria.

**A. Net reclassification related to risk determined from ACC/AHA versus NICE guideline**

| **Outcome: No Obstructive Disease** | | | | |
| --- | --- | --- | --- | --- |
|  |  | **NICE Risk Classification** | | |
|  |  | Low | Intermediate-high | % Reclassified |
| **ACC/AHA Risk Classification** | Low | 275 | 14 | 5 |
|  | Intermediate-high | 677 | 4298 | 14 |
| **Outcome: Obstructive Disease** | | | | |
|  | | **NICE Risk Classification** | | |
|  |  | Low | Intermediate-high | % Reclassified |
| **ACC/AHA Risk Classification** | Low | 12 | 0 | 0 |
|  | Intermediate-high | 83 | 801 | 9 |
|  | NRI (no-event): 0.126 (95% CI 0.116 to 0.136)  NRI (event): -0.093 (95% CI -0.113 to -0.073)  NRI (overall): 0.033 (95% CI 0.012 to 0.0541) | | | |

**B. Net reclassification related to risk determined from ESC versus NICE guideline**

| **Outcome: No Obstructive Disease** | | | | |
| --- | --- | --- | --- | --- |
|  |  | **NICE Risk Classification** | | |
|  |  | Low | Intermediate-high | % Reclassified |
| **ESC Risk Classification** | Low | 325 | 70 | 18 |
|  | Intermediate-high | 627 | 4242 | 13 |
| **Outcome: Obstructive Disease** | | | | |
|  | | **NICE Risk Classification** | | |
|  |  | Low | Intermediate-high | % Reclassified |
| **ESC Risk Classification** | Low | 18 | 0 | 0 |
|  | Intermediate-high | 77 | 801 | 9 |
|  | NRI (no-event): 0.106 (95% CI 0.096 to 0.116)  NRI (event): -0.086 (95% CI -0.105 to -0.067)  NRI (overall): 0.020 (95% CI -0.001 to 0.041) | | | |

**Supplementary Table 2: Net reclassification improvement across both trial cohorts.**

ACC/AHA, American College of Cardiology/American Heart Association; ESC, European Society of Cardiology; NICE, National Institute of Health and Care Excellence; NRI, net reclassification improvement; CI, confidence interval.
